# Inhibition of FABP4 attenuates cardiac fibrosis through inhibition of NLRP3 inflammasome activation

**DOI:** 10.22038/IJBMS.2022.64499.14186

**Published:** 2022-10

**Authors:** Xi Zhu, Xiaogang Zhang, Xinpeng Cong, Luoning Zhu, Zhongping Ning

**Affiliations:** 1 Department of Cardiology, Shanghai University of Medicine & Health Sciences affiliated Zhoupu Hospital, Shanghai 201318, China

**Keywords:** Cardiac fibrosis, Collagen, FABP4, Fibroblast, MMP-2, MMP-9, NLRP3 inflammasome

## Abstract

**Objective(s):**

Cardiac fibrosis is a key biological process of cardiac remodeling and heart failure. Fatty acid-binding protein 4 (FABP4) is a lipid-binding protein that can regulate glucose and lipid homeostasis, and its expression was elevated in heart failure. However, whether FABP4 is involved in cardiac fibrosis remains unknown.

**Materials and Methods:**

The cardiac fibrosis model was established in male C57BL/6 mice with subcutaneously infused angiotensin II (Ang-II) (2.8 mg/kg/day) for 4 weeks. DMSO or FABP4 inhibitor BMS309403 (50 mg/kg/day) was intraperitoneally injected for 4 weeks. Ang II-infused mice, FABP4 inhibitor (BMS309403) injected mice, and ventricular tissue were used for morphological studies, and histological and biochemical analyses (FABP4 protein composition and expression).

**Results:**

Ang II infusion increased FABP4 mRNA and protein expression in the mouse ventricular tissue. After treatment with FABP4 inhibitor BMS309403 for 4 weeks, mice showed improved cardiac structure and function as detected by echocardiography. BMS309403 suppressed cardiac and systemic inflammatory response, reduced collagen deposition, and mRNA expression of collagen type I (COL1A1) and collagen type III (COL3A1) in Ang II-infused mice. BMS309403 also reduced the number of α-smooth muscle actin (α-SMA)+cells and decreased the mRNA expression of α-SMA, matrix metalloproteinases-2 (MMP-2), MMP-9, and transforming growth factor-β (TGFβ) in ventricular tissue.

**Conclusion:**

The inhibitory effect of BMS309403 on cardiac fibrosis might be associated with inhibition of NLRP3 inflammasome activation, which Ang II activated. Thus, our data speculated that inhibition of FABP4 could significantly induce cardiac fibrosis.

## Introduction

Cardiac fibrosis is a pathological extracellular matrix (ECM) remodeling process. The condition is caused due to disrupting cardiac morphology, abnormal ECM accumulation, and impaired heart muscle function (1-3). Previous studies reported that ECM deposition could be a protective and beneficial mechanism for inflammation healing and tissue regeneration. However, excessive and spontaneous ECM deposition, specifically collagen type I secretion, may lead to impaired tissue function (4).

In cardiac fibrosis, inflammatory response plays a crucial role. Nucleotide-binding domain and leucine-rich repeat-containing family, pyrin domain-containing-3 (PYD-3), or Nod-like receptor protein 3 (NLRP3) inflammasome are an essential definitive containing NLRP3 receptor, an apoptosis-associated speck-like protein consisting of a caspase recruitment domain (ASC) and precursor caspase-1 (5, 6). NLRP3 inflammasome is well characterized by its pathophysiological activities and clinical implications (7, 8). Unusually, it can be activated by different stimuli, including nigericin (*Streptomyces* *hygroscopicus* secreted, an antibiotic) or ATP released from damaged cells (7). However, the un-regulated NLRP3 activation could lead to unconstrained infections, neurodegenerative diseases, autoimmune diseases, metabolic disorders, and other human disorders (9). Therefore, the interaction between NLRP3 and stimuli, and thus the mechanism by which NLRP3 is stimulated, remains unknown.

Fatty-acid binding protein 4 (FABP4) is a member of the FABP family and was first detected in mature adipocytes and adipose tissue, and is closely related to inflammation (10, 11). FABP4 plays a crucial role in insulin resistance, diabetes mellitus (type 2), gestational diabetes, and other metabolic syndromes (12-15). It has been further reported that FABP4 is associated with atherosclerosis and cardiovascular diseases (16, 17). Recent studies reported that FABP4 mRNA expression is higher in heart failure patients than in normal patients and is linked with heart failure severity. Thus, FABP4 is associated with heart failure (18). This evidence indicates that inhibition of FABP4 may attenuate cardiac fibrosis.

Our study identified FABP4 as a potential regulator of cardiac fibrosis because the expression of FABP4 was increased in the serum of cardiac fibrosis and Ang II-treated experimental mice. The results indicated that FABP4 inhibitor BMS309403 improved cardiac structure and function, attenuated cardiac and inflammatory response, and decreased collagen deposition and mRNA expression in Ang II-infused mice. BMS309403 also reduced the number of α-SMA+cells and reduced the mRNA expression of α-SMA, MMP-2, MMP-9, and TGFβ in ventricular tissue. The above results speculated that FABP4 could be a possible target for the treatment of cardiac fibrosis as well as a diagnostic biomarker of cardiac fibrosis.

## Materials and Methods

***Animals and treatment***

Male C57BL/6 mice (8-9 weeks old, 24-26 g) were used to establish the cardiac fibrosis model. This study was approved by the Animal Care and Use Committee of our Hospital. All experimental procedures were conducted following the Guide for the Care and Use of Laboratory Animals. Mice were infused with saline or Ang II to induce cardiac fibrosis by osmotic mini-pumps (2.8 mg/kg/day) for 4 weeks. For FABP4 inhibitor treatment, mice were intraperitoneally injected with either DMSO or BMS309403 (50 mg/kg, once a day) (HY-101903, Med Chem Express) for 28 consecutive days.

***Echocardiography***

After 4 weeks of Ang II infusion and BMS309403 administration, mice underwent echocardiography (Vevo 3100 Imaging System) to assess the cardiac structure and function. Mice were anesthetized with 2% isoflurane and placed on a warming pad. Electrocardiogram (ECG) was used to monitor the heart rate and rhythm. Transthoracic echocardiography was performed, and Doppler echocardiographic images were taken at the mid-papillary level with a 30 MHz transducer. LV mass and LVID were measured using a two-dimensional M-mode in the parasternal short-axis view. Cardiac output (CO) was calculated as follows: cardiac output=(LVIDd-LVIDs)×heart rate, where LVIDd and LVIDs represent LV internal diameter in diastole and systole, respectively.

***Histology and immunohistochemistry***

Mice were anesthetized with pentobarbital sodium, decapitated, and the ventricular tissue was fixed with 4% paraformaldehyde, paraffin-embedded, and cut serially into sections (thickness 5 μm). Tissues were ascended on glass slides and deparaffinized. Slides were stained with hematoxylin-eosin for histology and Masson’s trichrome for collagen fiber. For immunohistochemistry, slides were incubated with rabbit antibodies against FABP4 (ab92501; Abcam, UK) or α-SMA antibody (ab5694; Abcam, UK) at 37 ^°^C, followed by biotinylated HRP conjugated secondary antibody. The slides were washed and counter-stained. Images were photographed (×200 magnification). The collagen volume fraction was measured and expressed as the ratio of the connective tissue area to the total tissue area that was averaged from 10 images.

***Measurement of serum cytokines***

Enzyme-Linked Immunosorbent Assays (ELISA) determined the cytokine levels in the serum of mice. The blood sample was obtained from the caudal vein of mice after 4 weeks of Ang II infusion, and serum was isolated and stored at -80 ^°^C. Levels of TNF-α (MTA00B), IL-6 (M6000B), and TGF-β (DB100B) were measured with commercial ELISA kits (R&D Systems, MN, USA).

***Real-time quantitative polymerase chain reaction (RT-qPCR)***

Total RNA was extracted from ventricular tissue with TRIzol reagent (Invitrogen, USA) and was reversely transcribed into complementary DNA (cDNA), and the genomic DNA was digested with DNase I (TaKara Bio, China). mRNA was amplified by RT-qPCR using ABI PRISM 7500 Fast sequence detection system (Applied Biosystems, CA, USA). The 2-ΔΔCt method was used to calculate the mRNA levels of each gene after normalizing to GAPDH. The primer sequences were used in this study listed in Table 1.

***Western blot analysis***

Proteins were extracted from ventricular tissue and quantified. Total protein (50 μg) was separated by SDS-PAGE and transferred onto PVDF membranes. With 5% skimmed milk, the membranes were blocked and incubated with primary antibodies against FABP4 (1:500; ab92501; Abcam, UK), NLRP3 (1:200; ab214185; Abcam, UK), ASC (1:200; ab155970; Abcam, UK), pro-caspase-1 and cleaved caspase-1 (1:500; ab207802; Abcam, UK), and GAPDH (ab9485; Abcam, UK). Then followed by incubation with a secondary antibody (goat anti-rabbit, 1:2000; 1:2000; ab7090; Abcam, UK). Protein bands were identified with ECL reagents (Amersham Biosciences, UK). GAPDH was used as an internal control, and the protein bands were analyzed using Image-Pro Plus software.

***Statistical analysis***

Data were expressed as the mean±SD and were analyzed using the SPSS software package (version 20.0, SPSS, Inc., USA). Comparisons among groups were analyzed using one-way ANOVA. A two-tailed t-test was used to compare two groups, and one-way ANOVA was used to compare three groups, followed by Student-Newman-Keuls tests. *P*<0.05 was considered statistically significant.

## Results

***FABP4 expression was elevated in the mouse heart after Ang II infusion***

**Figure 1:**
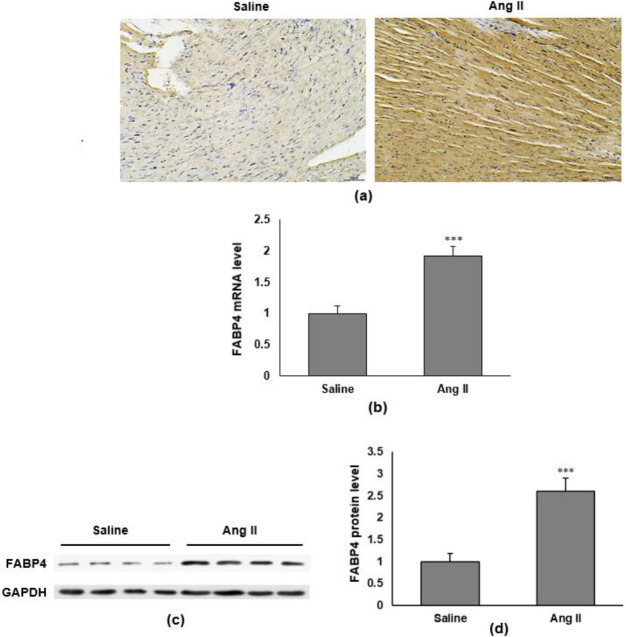
FABP4 expression is elevated in cardiac fibrosis mice. Mice were infused with Ang II (2000 ng/kg/min) for 4 weeks. (a) Representative images of FABP4 in the ventricular muscle of Ang-II-induced mice by immunohistochemistry. Magnification: ×200. (b) RT-qPCR detects FABP4 mRNA levels in the ventricular muscle of mice with and without Ang II infusion. (c) Representative protein bands of FABP4 in Ang-II-induced mice model. (d) Quantification of FABP4 protein levels (n=8 in each group). Data are presented as mean±SD. ***P<0.001 vs saline group

We first determined whether FABP4 was expressed in the mouse heart after Ang II treatment. Mice were infused with Ang II (2.8 mg/kg/day) for 4 weeks. Immunohistochemistry assay showed higher FABP4 expression in the cytoplasm and intercellular matrix of ventricular muscle (Figure 1(a)). To investigate the transcription of FABP4, we measured FABP4 mRNA levels in the mice model. RT-qPCR showed increased FABP4 mRNA in the ventricular muscle of mice after Ang II infusion (*P*<0.001) (Figure 1(b)). To determine whether the protein expression of FABP4 is also changed, we performed Western blotting to determine the protein levels of FABP4 (Figure 1(c)). The FABP4 protein expression was significantly higher in Ang-II-infused mice’s ventricular muscle than in the saline-infused mice (Figure 1(d)). These results indicate that Ang II regulates FABP4 expression at both mRNA and protein levels.

***Effect of FABP4 inhibitor on LV structure and function in Ang-II-infused mice***

Mice were infused with Ang II and intraperitoneally injected with FABP4 inhibitor BMS309403 (50 mg/kg, once a day) for 4 weeks. Echocardiography was performed to show the structure and function of the left ventricle (LV) (Table 2). Bodyweight (BW) and heart rate (HR) were not different between untreated saline and Ang II mice. BMS309403 did not alter body weight and heart rate. Ang II caused a significant increase in LV mass than the saline group, which decreased by BMS309403 (*P*<0.001). Ang II also increased left ventricular internal diameter (diastole) (LVIDd) and left ventricular inner diameter (systole) (LVIDs), and these changes were significantly reversed by BMS309403 (both *P*<0.05). Moreover, the cardiac function was also significantly changed, and the cardiac output (CO) was increased by Ang II but reduced by BMS309403 (*P*<0.05).

***FABP4 inhibitor suppressed Ang II-induced inflammatory response***

**Figure 2:**
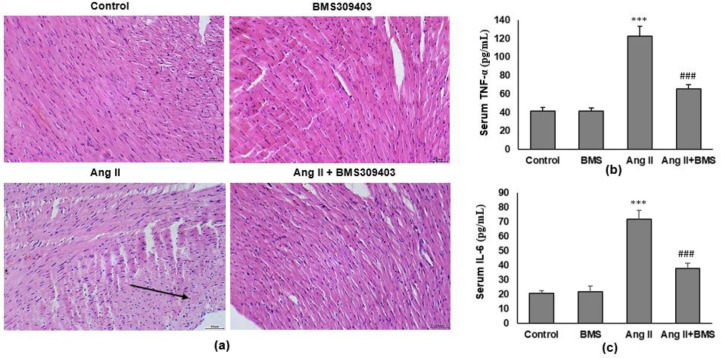
FABP4 inhibitor suppresses Ang II-induced inflammatory response of cardiac tissue. Mice were infused with Ang II and intraperitoneally injected with FABP4 inhibitor BMS309403 (50 mg/kg, once a day) for 4 weeks. (a) Representative images of the ventricular tissues stained with hematoxylin-eosin (H&E). The arrow indicates the infiltrated inflammatory cells. Magnification: ×200. Systematic inflammation was evaluated by measurement of the serum inflammatory cytokines using ELISA, including (b) TNF-α and (c) IL-6 (n=8 in each group). Data are presented as mean ± SD

We stained the ventricular tissue with HE to determine whether the FABP4 inhibitor altered inflammation in the heart. Compared with saline-infused mice, Ang II–infused mice showed increased inflammatory cell infiltration in the ventricular tissue of mice. The inflammatory cell infiltration was further attenuated by BMS309403 treatment (Figure 2(a)). To ascertain whether inflammatory cell infiltration in ventricular tissue is associated with systemic inflammation, we additionally measured the serum levels of two proinflammatory cytokines, TNF-α and IL-6. Ang II significantly increased serum levels of TNF-α and IL-6. However, these changes were markedly attenuated by BMS309403 (both *P*<0.001) (Figure 2(b-c)).

***FABP4 inhibitor attenuated Ang II-induced cardiac fibrosis***

**Figure 3:**
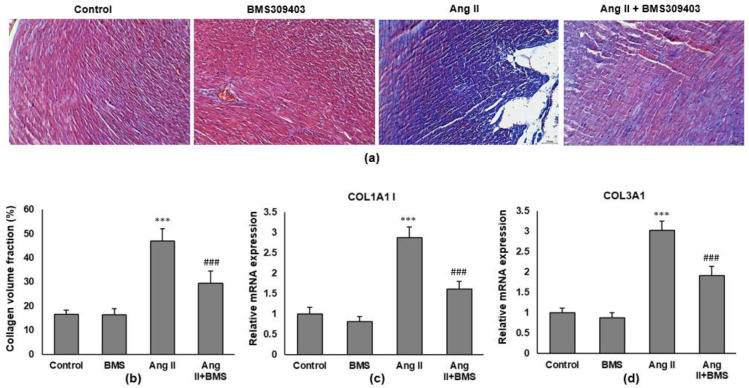
FABP4 inhibitor reduces cardiac fibrosis in Ang II-infused mice. (a) Ventricular tissues were stained with Masson’s trichrome to show the collagen fiber (blue in the interstitium). Magnification: ×200. (b) Quantification of the fibrotic area. RT-qPCR was carried out to determine the mRNA expression of collagen gene, (c) COL1A1 (encode collagen I), and (d) COL3A1 (encode collagen III). Data are presented as mean±SD, N=8

Cardiac fibrosis was evaluated by Masson’s staining of ventricular tissue. Ang II leads to a significant increase in myocardial interstitial fibrosis, which could be markedly attenuated after BMS309403 treatment (Figure 3(a-b)). Consistent with this, the mRNA expression of fibrosis markers (collagen I and collagen III) was reduced in the hearts of mice treated with BMS309403 as assessed by RT-qPCR (Figure 3(c-d)).

***FABP4 inhibitor reduced the fibroblast activation and suppressed the TGFβ pathway***

**Figure 4:**
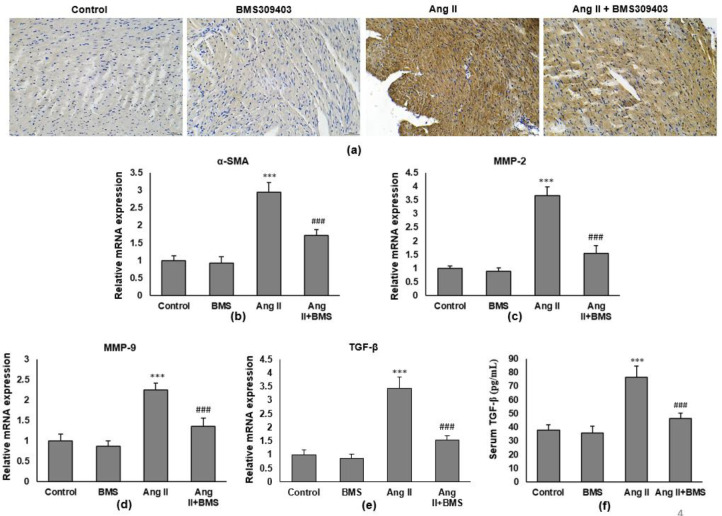
FABP4 inhibitor suppresses expression of genes associated with the TGF-β pathway. (a) Immunohistochemistry was carried out, and representative images of α-SMA are shown. Magnification: ×200. RT-qPCR was carried out to determine the mRNA expression of (b) α-SMA, (c) MMP-2, (d) MMP-9, and (e) TGF-β. All mRNA levels are normalized to GAPDH mRNA. (F) ELISA was carried out to measure the serum TGF-β level of mice. n=8 in each group

 To investigate if FABP4 modulates fibroblast activation, we performed immunohistochemistry to evaluate the number of fibroblast in the intercellular matrix. FABP4 inhibitor markedly attenuated Ang II-induced increase in the number of α-SMA-positive cells, indicating that FABP4 inhibition can suppress the transformation of fibroblasts into myofibroblasts (Figure 4(a)). Moreover, RT-qPCR showed that Ang II infusion significantly increased the mRNA expression of α-SMA (Figure 4(b)), MMP-2 (Figure 4(c)), MMP-9 (Figure 4(d)), and TGFβ (Figure 4(e)), which were all significantly attenuated by BMS309403. This FABP4 inhibitor also reduced serum TGFβ concentration in Ang II-infused mice as assayed by ELISA (Figure 4(f)). The results indicated that the TGFβ pathway is involved in the inhibitory process of cardiac fibrosis by FABP4 inhibition.

***FABP4 inhibitor modulated the expression of proteins associated with NLRP3 inflammasome***

**Figure 5:**
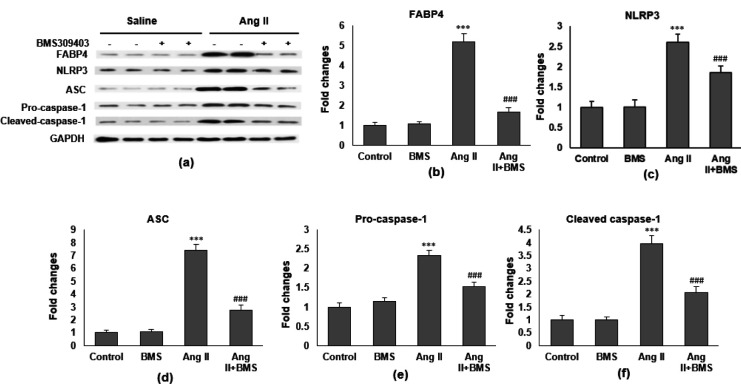
FABP4 inhibitor suppresses the NLRP3 inflammasome activation. (a) Representative western blot bands of FABP4 and NLRP3 pathway proteins, including (b) FABP4, (c) NLRP3, (d) ASC, (e) pro-caspase-1, and (f) cleaved caspase-1 (n=8 in each group)

Western blot was performed to measure the protein level of NLRP3 inflammasome-related proteins (Figure 5a). FABP4 inhibitor reduces the FABP4 protein expression in ventricular tissue of Ang II-infused mice (Figure 5b). Ang II infusion increased protein expression in NLRP3, ASC, pro-caspase-1, and cleaved caspase-1, and this indicated that NLRP3 inflammasome activation is involved in the process of Ang II-induced cardiac fibrosis. FABP4 inhibitor treatment significantly reduced these protein expressions in Ang II-infused mice (Figure 5c-f).

**Table 1:** List of oligonucleotide primers used in this study

Genes	Forward primer (5′-3′)	Reverse primer (5′-3′)
FABP4	CATCAGCGTAAATGGGGAT	TCGACTTTCCATCCCACTTC
COL1A1	TAAAGGGTCATCGTGGCTTC	GACGGCTGAGTAGGGAACAC
COL3A1	CTGTAACATGAAACTGAACTGAAA	CCATAGCTGAACTGAAAACCACC
α-SMA	CTGTCCCTCTATGCCTCTGG	AGGGCTGTGATCTCCTTCTG
MMP-2	TCAACGGTCGGGAATACAGC	AGCTGTTGTAGGAGGTGCCCT
MMP-9	AAGGGTACAGCCTGTTCCTGGT	CTGGATGCCGTCTATGTCGTCT
TGF-β	AGCAACAATTCCTGGCGTTACCTT	CCTGTATTCCGTCTCCTTGGTTCAG
GAPDH	TGTGTCCGTCGTGGATCTGA	TTGCTGTTGAAGTCGCAGGAG

FABP4: Fatty acid binding protein 4; α-SMA: α-smooth muscle actin; MMP-2: Matrix metalloproteinases-2; MMP-9: Matrix metalloproteinases-9; TGF-β: Transforming growth factor-β; GAPDH: Glyceraldehyde-3-phosphate dehydrogenase

**Table 2:** Characteristics and echocardiography measurements of mice after 4 weeks Ang II infusion

Groups	Control	BMS309403	Ang II	Ang II+BMS309403
BW (g)	23.31±1.32	23.30±1.36	23.85±1.02	23.18±1.62
HR (bpm)	452.63±35.40	450.38±30.90	442.38±25.85	464.25±31.48
LV mass (mg)	75.65±4.62	76.74±5.93	99.47±6.63***	86.43±5.74###
LVIDd (mm)	3.54±0.31	3.58±0.19	4.02±0.21**	3.67±0.22##
LVIDs (mm)	2.47±0.19	2.57±0.11	3.05±0.36**	2.66±0.25#
CO (mL/min)	14.60±1.67	15.77±1.87	18.11±1.85**	15.51±2.23#

Data are represented as means±SD

**P<0.01, *** *P<*0.001 vs control group; ^#^*P<*0.01, ^##^*P<*0.01, ^###^*P<*0.001 vs Ang II group

BW: Body weight; HR: Heart rate; LV mass: Left ventricle mass; LVIDd: Left ventricular internal diameter (diastole); LVIDs: Left ventricular internal diameter (systole); CO: Cardiac output

***P<0.001 vs saline group; ^###^*P<*0.001 vs Ang II group

***P<0.001 vs saline group; ^###^*P<*0.001 vs Ang II group

***P<0.001 vs saline group; ^###^*P<*0.001 vs Ang II group

***P<0.001 vs saline group; ^###^*P<*0.001 vs Ang II group

## Discussion

In this study, we explored the effect of FABP4 on cardiac fibrosis. The results showed that FABP4 expression was improved in the cardiac fibrosis patient’s serum and Ang II-infused mice, while inhibition of FABP4 reversed these effects. BMS309403 is a potent, targeted FABP4 inhibitor that interacts with fatty acid binding within a protein and inhibits endogenous fatty acid binding through competitive inhibition. By modulating the macrophage-myofibroblast transition, BMS309403 suppresses FABP4 and reduces renal interstitial fibrosis (19). In the current study, BMS309403 potentially enhanced cardiac structure and function and reduced cardiac and inflammatory response, reduced collagen deposition and mRNA expression in Ang II-treated mice. In addition, BMS309403 decreased the number of α-SMA+cells and decreased the mRNA expression of α-SMA, MMP-2, MMP-9, and TGFβ in ventricular tissue. Thus, the results demonstrate that FABP4 could be a potential therapeutic target of cardiac fibrosis.

An important mediator of hypertension and heart failure (HF) is angiotensin II (Ang II), the main effector of the renin-angiotensin system (RAS). Chronic RAS activation is linked to the development of HF, and chronic Ang II leads heart’s structural and electrical remodeling (20). In this experiment, mice were treated with intraperitoneal injections of the FABP4 inhibitor BMS309403 (50 mg/kg, once a day) along with Ang II infusions. To visualize LV’s anatomy and function, echocardiography was used. Between untreated saline and Ang II mice, there were no differences in BW and HR. BMS309403 did not change the body mass index and pulse rate. Ang II significantly increased LV mass compared with the saline group, which had a BMS309403 (P0.001) reduction. Both LVIDd (diastole) and LVIDs (systole) were considerably decreased by BMS309403 (both P 0.05) in response to Ang II. The cardiac function was also considerably altered, and Ang II enhanced cardiac output (CO), but BMS309403 decreased it (*P*<0.05).

Cardiac fibrosis is a common pathological state in different cardiac diseases, characterized by abnormal accumulation of ECM and proliferation of cardiac fibroblasts in the heart (21-22). Cardiac fibroblasts are stimulated and segregated into myofibroblasts that migrate and accumulate in the inflamed areas of the myocardium. They can be expanded to the nearby in most cardiac pathologic conditions, generating collagens, specifically primarily collagens I (Col I) and III (Col III), and other ECM (23). Fibrotic cardiac tissue is rigid and low compliance due to lacking the ability to lead impulses and contract, which can be impaired cardiac function consequence of heart failure (21, 23-24). FABP4 is considered a biomarker for cardiac fibrosis, while the FABP4 level improved in cardiac fibrosis patients may speculate the association of FABP4 in myocardial fibrosis and the advancement of cardiac fibrosis. Previous studies stated that the risk of atrial fibrosis could be associated with the severity of heart failure and decreased response to catheter ablation (25-26). Thus, FABP4 could be a potential target for the treatment of cardiac fibrosis patients, as well as a diagnostic biomarker of cardiac fibrosis.

Fatty-acid binding protein 4 (FABP4), also known as adipocyte FABP (A-FABP) or aP2, belongs to an intracellular lipid chaperone family that is revealed in both adipocytes and macrophages (27). It has a crucial role in the development of insulin resistance, atherosclerosis, and cardiovascular diseases (12, 16). FABP4 may have been directly linked to cardiac alteration. It has a positive correlation in both LV dysfunction, and myocardial perfusion abnormalities were found in coronary artery patients (28). However, recent studies reported that inhibition of FABP4 has a potentially significant effect against diabetes mellitus and atherosclerosis (29). Researchers found that individuals with cardiac fibrosis had higher FABP4 levels in their ventricular tissue and serum and that FABP4 levels were related to cardiac dysfunction and heart failure biomarker levels. Our study found that serum FABP4 was significantly higher in cardiac fibrosis mice than those without cardiac fibrosis mice, speculating that FABP4 may be associated with the pathogenesis of cardiac fibrosis.

Transforming growth factor beta-1 (TGF-β1) is the potential fibrogenic factor. It is a cytokine that plays an essential role in mesenchymal transition (EMT) progression. TGF-β1-induced EMT plays a critical role in cardiac fibrosis and hypertrophy (30-31). Our study investigated if the inhibition of FABP4 reduces fibroblast activation by inhibiting TGF-β1 and reported that FABP4 inhibition significantly inhibited the transformation from fibroblast to myofibroblasts (Figure 4a). The inhibition of FABP4 also decreased serum TGFβ concentration in Ang II-treated mice as assayed by ELISA (Figure 4f). The study results suggested that the TGFβ pathway may involve attenuating cardiac fibrosis through inhibition of FABP4.

The inflammasome is a multiprotein complex that is mediated by immune responses to inflammation-inducing stimuli, including cellular damage and pathogen infection (32). NLRP3 inflammasome contains NLRP3, ASC, and caspase-1 (33). With respect to stimuli, NLRP3 stimulates inflammasome assembly and recruits pro-caspase-1, which leads to its cleavage and activation. Activated caspase-1 further cleaves pro-IL-1β and pro-IL-18 to mature active IL-1β and IL-18, respectively (32-33). Currently, it has been indicated that FABP4 regulates NLRP3 inflammasome activation in macrophages (34). Consistently, FABP4 was associated with NLRP3 inflammasome activation and enhanced the production of cytokines (IL-17A and IL-23) in primary macrophages. Moreover, IL-17A was involved in accelerating atherosclerosis via enhancing FABP4-mediated endoplasmic reticulum (ER) stress in macrophages (35). Our study showed that Ang II treated mice improved protein expression in NLRP3, ASC, pro-caspase-1, and cleaved caspase-1. The results speculated that NLRP3 inflammasome activation could be involved in the process of Ang II-induced cardiac fibrosis. Thus, inhibition of FABP4 significantly attenuated NLRP3 inflammasome protein expressions in Ang II-infused mice (Figure 5c-f). 

## Conclusion

FABP4 expression is increased in the serum of cardiac fibrosis mice, which exacerbates fibrosis and inflammatory cytokine production. In contrast, inhibition of FABP4 improved cardiac structure and function, restrained cardiac and systemic inflammatory response, and attenuated collagen deposition and mRNA expression of COL1A1 and COL1A3 in Ang II-infused mice through inhibition of NLRP3 inflammasome activation. Our results suggested that FABP4 could be a potential therapeutic target for the treatment of cardiac fibrosis, as well as a diagnostic biomarker of cardiac fibrosis.

## Authors’ Contributions

NZ designed and supervised the project and revised the manuscript. Zhu X and WY performed experiments and wrote the first draft of the manuscript. Zhang X, CX, and ZL analyzed the data and performed statistical analysis.

## Availability of data and materials

The datasets used and/or analyzed during the current study are available from the corresponding author upon reasonable request.

## Conflicts of Interest

The authors declare no conflicts of interest with other people or organizations.
